# The impact of day care attendance on antibiotic use among children aged 0–12 years: A population-based register study

**DOI:** 10.1371/journal.pone.0335354

**Published:** 2025-11-17

**Authors:** Tapio Räsänen, Miia Saarikallio-Torp, Hanna Rättö, Heini Kari

**Affiliations:** 1 Research Unit, The Social Insurance Institution of Finland (Kela), Helsinki, Finland; 2 INVEST Research Centre, University of Turku, Turku, Finland; Inonu University, Faculty of Pharmacy, TÜRKIYE

## Abstract

Day care attendance is known to be an important source of infection transmission in children. Having older siblings has also been shown to increase the risk of recurrent infections. We use comprehensive register data to study if the cumulative antibiotic use in children differs according to the duration of how long the child has been in home care before entering day care. The study focuses on children born in Finland between 2000 and 2005, with follow-up until age 12. Children are linked to their biological parents, and the analysis includes control variables such as mother’s age and education level. In addition to descriptive evidence, we employ statistical models to study the association between the duration of home care and antibiotic exposure. The results show that almost all children were exposed to prescribed antibiotics within the first 12 years of their life. One fifth of the children had more than 15 antibiotic purchases by the age of 12. Four most commonly prescribed antibiotics were amoxicillin, azithromycin, cefalexin, and amoxicillin with beta-lactamase inhibitor. The results from Poisson regression model affirmed that cumulative use was lower in children with longer home care duration. Regardless of the number siblings, the number of cumulative antibiotic purchases remained lowest in the group of children with the longest home care period, and the results remained robust after controlling for the mother’s age and education, and immigrant background. These findings contribute to a broader understanding of how early childhood care arrangements may be associated with long-term patterns of antibiotic use, with potential relevance for public health planning and antimicrobial stewardship.

## Introduction

Respiratory tract infections (RTI) are common infections in childhood, with an average of six to eight episodes a year [1,2]. Children’s infections lead to absences from day care and school, as well as parental absences from work. RTIs cause discomfort, but in most cases, the symptoms will resolve within 1–2 weeks without treatment. Viral RTIs, such as the common cold, should not be treated with antibiotics. However, ear infections, sinusitis, and pneumonia are bacterial complications of RTI, in which antibiotics are often used in children.

Antimicrobial medicines are the cornerstone of modern medicine [3]. In Finland, guidelines exist for rational antibiotic prescribing in children, yet deviations, particularly in outpatient settings where most antibiotics are prescribed, have been reported [4,5]. This may increase the risk of antimicrobial resistance (AMR), which is an increasingly serious threat to global public health. AMR occurs when bacteria, viruses, fungi, and parasites no longer respond to antimicrobial medicines and infections become difficult, or even impossible, to treat, which increases the risk of disease spread, severe illness, disability, and death [3]. AMR is directly related to how antibiotic medications are used in society. In Finland, previous studies have shown a decreasing trend in the prescribing of antibacterial medicines for children before the COVID-19 (coronavirus disease) pandemic in 2020 [4,6,7]. A further reduction was observed after the COVID-19 pandemic [8], paralleling the findings from the whole European Economic Area (EEA) [9]. However, despite the decrease seen in some countries, the worldwide consumption of antibiotics has been increasing [10].

In addition to population-level risks related to the rise of AMR, inappropriate use of antimicrobials in children can be associated with other increased risks. A recent systematic review and meta-analysis showed that antibiotic exposure in children is associated with, for example, an increased risk of atopic dermatitis, allergic symptoms, food allergies, asthma, obesity, psoriasis, autism spectrum disorders, and neurodevelopmental disorders [11]. There are also studies that have reported a possible association between early-life antibiotic exposure and type 1 diabetes [12,13]. Studies suggest that antibiotic exposure plays an important role in the development of childhood immune disorders, likely through disruption of the microbiota during early life [14]. It has been estimated that 10% of children account for 25% of the total antibiotic use [15].

Previous studies have shown that socioeconomic and other family-related factors can be related to the levels of respiratory tract infections and antibiotic exposure in children. Thrane et al. (2003) [16] and Jensen et al. (2016) [15] showed that higher parental educational level was associated with a lower number of antibiotic treatments, whereas a study by Hatakka et al. (2010) [17] found that mother’s academic education increased the risk of recurrent acute respiratory illnesses. Using a socioeconomic measure based on the region of residence, Larsen et al. (2021) [18] found a declining association between socioeconomic municipality scores and antibiotic treatments, indicating that regions with a lower socioeconomic status were associated with higher number of antibiotic treatments. Regarding other family-related factors, Hatakka et al. (2010) [17] found that furry pets and older child age reduced the risk of recurrent acute respiratory illnesses, and Toivonen et al. 2016 [19] showed that having older siblings increase the risk of recurrent infections. Thus, although some previous findings have been contradictory regarding the direction of the association, it nevertheless appears that the child’s background is associated with antibiotic exposure.

Day care attendance is known to be an important source of infection transmission in children [20,21]. Previous studies have observed a rapid increase in respiratory infections and antibiotic use after starting day care [16,22,23], followed by a subsequent decrease [22]. Thus, factors affecting day care attendance also play a role in the number of respiratory infections and antibiotic use in children.

In Finland, the day care attendance rate has been lower than in the other Nordic countries and also below the OECD (The Organisation for Economic Co-operation and Development) average, especially for younger children – apart from the most recent years [24,25]. The lower attendance rate is likely linked to the Finnish family benefit system, in which families are entitled to child home care allowance (HCA) if the child does not attend day care. This, in turn, enables parents to take care of the child at home. The HCA is widely used (90% of mothers), and most of the families use it at least for some time after the parental leave period (mean duration 13 months) [26]. At the time of the study data, in the Finnish parental leave scheme, children were approximately 10 months old when the earnings-based parental leave period ended.

In this paper, we use comprehensive register data to describe the antibiotics used by the study group, and to study if the cumulative antibiotic use differs between children by the duration of home care before entering day care. We hypothesise that longer duration of home care (i.e., delayed entry into day care) is associated with lower cumulative antibiotic use. We use descriptive methods and Poisson models to explore the hypothesis, accounting also the socioeconomic and other family-related factors. To be able to account for the potential impact of school entry at age 7 in Finland, the follow-up lasted until the children were 12 years old. As antibiotic exposure and strategies for taking care of young children are likely to be linked, it is important to study their associations. The findings may provide tools for enhancing the rational use of antibiotics.

## Materials and methods

### Institutional context of childcare leaves in Finland

As a Nordic welfare state, Finland provides universal parental leave benefits to all parents after the child is born [27]. In addition, high-quality, subsidised early childhood education and care (ECEC) services are provided to all children under the age of seven by municipalities [28]. In Finland, children start primary school at the age of seven years. Before that, pre-primary education (for six-year-old children) is available (mandatory since 2015).

Children are usually approximately 10 months old when the parental leave period ends [26]. If the child does not attend ECEC (hereafter day care) after the parental leave period, families are entitled to HCA. This enables parents to take care of a child at home until the child reaches the age of three years. Families are entitled to HCA also for their other children below school age (not attending day care) if the youngest child is under the age of three and not attending day care.

Childcare fees for day care services are heavily subsidised by the government, and day care is free of charge for low-income families, as the fees are means-tested by family income and number of persons in the household [29]. The maximum fees are set at the state level, with the same maximum amounts applied across all municipalities. The quality of day care services is considered to be rather high in the Scandinavian countries, including Finland, with skilled staff and high adult-to-child ratios as well as generally smaller group sizes [30,31]. In Finland, the maximum number of children per adult is regulated by law and is currently 1:4 for children under the age of three and 1:7 for children aged three or older. However, the actual group sizes are decided locally at the municipal level [32]. Largely due to the subsidized and widely available day care as well as the HCA for the children under the age of three, informal childcare (e.g., grandparents, relatives, friends) is notably rare in Finland [33].

### Data

#### Study sample.

Our sample was drawn from the Social Insurance Institution of Finland’s (Kela) registers and included live children born in Finland between 2000 and 2005. The data a was 70% simple random sample of Finnish women who gave birth between 2000 and 2005. We followed the children until the end of 2017, or until the year they turned 12 years of age. The age is defined as the child’s age at the end of the inspected year.

#### Antibiotic utilisation.

The national medicine reimbursement scheme covers the majority of the prescribed antibiotics in outpatient care. Using the register of reimbursed medicine purchases maintained by Kela, we retrieved data on antibiotic medications belonging to Anatomical Therapeutic Chemical (ATC) Classification J01 (Antibacterials for systemic use) and its substance subgroups dispensed between 2000 and 2017 for children included in the study. This study focused on systemic antibiotics, and data on topical antibiotics, such as eye drops or creams, were not included.

#### Duration of home care.

Instead of attending day care, children can also be cared for at home. We used individual-level HCA information to deduce a reverse measure for day care attendance, as national individual-level information on day care attendance has not been available in Finland until the 2020s.

In order to deduce a child’s day care attendance from HCA payments, we applied a novel method developed by Andresen et al. (2019) [34] and Räsänen and Österbacka (2024) [35]. For each child in our sample, we calculated the duration of home care after the parental leave period based on the total number of months the parents received HCA for the child. If no HCA was paid, the child was assumed to be in day care. Thus, the child’s participation in day care after the parental leave period was determined based on home care allowance use. As the HCA is widely used in Finland, approximately 90 percent of families use it [26], it serves as a rather good proxy for the day care attendance.

We retrieved data on HCA payments from Kela’s register. The register includes information on families eligible for HCA as well as whether older siblings in the family are also cared for at home. Families can receive HCA for a child under the age of three who does not attend day care. In addition, HCA is paid for older siblings aged 3–6 if they do not attend day care and have younger siblings under the age of three who are cared for at home.

It should be noted that the duration of home care does not include the parental leave period. As the average duration of parental leave is approximately 10 months, in most cases, the children are approximately 10 months old at the start of the home care period. For the analysis, we categorised the children into five groups based on the duration of HCA after the parental leave period. The categories were 0 months (only day care), 1–12 months, 13–24 months, 25–36 months, and 37 months or longer. Children for whom HCA was paid for 13–24 months (i.e., children aged approximately 22–34 months) were used as the reference group in the regression analysis.

#### Family characteristics.

We used Statistics Finland’s FOLK basic research data modules, which include longitudinal register-based information on sociodemographic factors covering all permanent residents in Finland [36]. First, we linked children to their biological parents and then included the following control variables used in the analysis: mother’s age (in full years on the last day of the year) and the number of biological children, as well as level of education (highest qualification/degree at the time of childbirth), and immigrant background. Children missing from the FOLK data set (e.g., children who have emigrated from Finland) and children who died during the follow-up period were excluded.

### Methods

First, we used descriptive methods to study antibiotic purchases for children during the whole study period (2000–2017). We analysed the shares of 0–12-year-old children with antibiotic purchases in one-year age categories and provided a description of the purchased antibiotics by ATC category. For the child’s first 12 years, we calculated and described the average cumulative number of antibiotic purchases based on the duration of HCA.

In addition to providing descriptive evidence, we employed statistical models to study the association between the number of HCA months and antibiotic exposure. Specifically, we examined the total exposure to antibiotics until the age of 12. In adjusted models, we accounted child’s birth order and mother’s age and education at child’s birth. We used Poisson regressions to estimate the following model:


$$\text{log}\left(\lambda_{i}\right)=\beta_{\text{0}}+X_{i}\beta +H_{i}\beta_{H}$$

where our response of interest $\lambda_{i}$ was cumulative purchases of antibiotics between the ages of 0 and 12. Our main explanatory variable was $H_{i}$ that is the sum of HCA months when the child was 0–6 years (0–72 months) of age. We set the reference category as 13–24 months since this corresponds to the maximum duration of home care for a single-child family. In fully adjusted models, we included a vector $X_{i}$ for control variables (child’s birth order and mother’s immigrant background, age and education at child’s birth). Since prescription patterns may have evolved over time, we used child’s birth year in all models, both unadjusted and adjusted models. Furthermore, we use separate models to examine the association by the number of siblings at age 12.

We conducted all statistical analyses using R (version 4.3.1).

### Ethics statement

As the study was based only on administrative, secondary register data, and no human subjects were contacted to collect the data, no Ethics Board approval was required [37] according to Finnish legislation. According to the General Data Protection Regulation of the European Union and the Finnish Data Protection Act, processing of personal data is permitted without informed consent of study subjects if the task is carried out in the public interest, such as scientific research. Kela and Statistics Finland approved the use of the data for the current study (decision number TK/2601/07.03.00/2021). The data used in the study were fully pseudonymised before the authors accessed the data, starting from 1 January 2024, via the FIONA remote access system, which is a secure environment for processing unit-level research data. All data preparation and linkage in the study were done with pseudo-identifiers, and the authors did not have access to information that could directly identify individual participants at any stage of the study. The study was conducted following good scientific practice, data protection guidelines and ethical standards. Legal restrictions prevent the open sharing of the pseudonymised data supporting the current study, as individual-level health data is considered highly sensitive and access is strictly regulated by law in Finland [38] (Act on secondary use of health and social data). Interested parties may apply for permission to access the data from Kela, https://www.kela.fi/web/en/data-permits-and-data-requests; email: tietoaineistot@kela.fi; and Statistics Finland, https://stat.fi/tup/tutkijapalvelut/ota-yhteytta_en.html; email: tutkijapalvelut@stat.fi.

## Results

First, we show descriptive evidence and statistics from the data, then we control for sociodemographic background variables using a Poisson regression model.

### Descriptive evidence

#### Use of antibiotics in all 0–12-year-old children.

**Fig 1:**
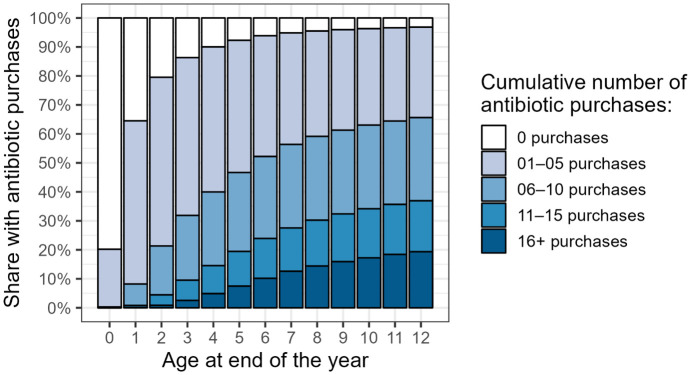
Share of children with cumulative number of antibiotic purchases from 0 to 12 years (2000–2017).

Fig 1 shows the number on antibiotic purchases in each of the one-year age categories and covers the entire study period. Each age category shows the cumulative number of antibiotic purchases, as they include all antibiotic purchases made thus far. By the age of 12, less than 5% of children had no outpatient antibiotic purchases and one fifth had more than 15 purchases, while the majority of the children had 1–10 antibiotic purchases.

**Fig 2:**
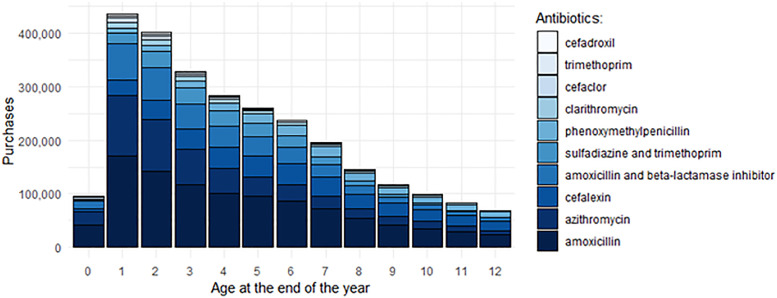
Ten most commonly purchased antibiotics by child’s age in 2000–2017.

The six most commonly prescribed antibiotics for 0–12-year-old children between 2000 and 2017 were amoxicillin, azithromycin, cefalexin, amoxicillin/beta-lactamase inhibitor, combination of sulfadiazine and trimethoprim, and phenoxymethylpenicillin. These pharmaceutical substances cover approximately 94% of the issued antibiotic prescriptions and are typically used to treat respiratory tract infections. Fig 2 shows the 10 most commonly purchased antibiotics by child’s age.

#### Use of antibiotics in 0–12-year-old children by the duration of HCA.

In Table 1, we show the descriptive sample characteristics by the duration of HCA, examined until the child’s 6th birthday. The average cumulative HCA was 21.4 months (just under 2 years). Thus, as the average parental leave is approximately 10 months, these children entered day care around the age of 2.5 years. The average age of the mother was 29.8 years at the child’s birth. Mothers were younger and lower educated in groups where the average duration of HCA was the highest. The average number of cumulative antibiotic purchases by the age of 12 was 10 purchases. In the groups of 1–12 months and 37 + months of HCA, the average numbers of cumulative purchases by the age of 12 were 10.3 and 8.6, respectively.

**Table 1:** Descriptive statistics of cumulative antibiotic purchases and background variables by duration of home care allowance.

	Duration of home care allowance (HCA)					
	0 months (No HCA)^1^	1–12 months	13–24 months	25–36 months	37 or more months	Average, all groups
**Child**						
Avg. cumulative antibiotic purchases until 6 years old, purchases	8.1	8.3	7.7	6.9	6.0	7.4
Median cumulative antibiotic purchases until 6 years old, purchases	7	7	6	5	4	6
Avg. cumulative antibiotic purchases until 12 years old, purchases	10.6	10.3	10.9	9.5	8.6	10.0
Median cumulative antibiotic purchases until 12 years old, purchases	9	8	9	7	7	8
Avg. home care allowance duration until 6 years old, months	0	7.0	18.4	28.5	47.8	21.4
Median home care allowance duration until 6 years old, months	0	7	18	28	47	21
First child (to the mother), %	47%	50%	50%	41%	46%	47%
**Mother**						
Avg. age (years)	30.8	30.1	29.5	30.3	28.1	29.8
Basic education, %	11%	10%	15%	20%	18%	15%
Secondary education, %	38%	35%	39%	44%	50%	41%
Tertiary education, %	52%	55%	46%	36%	32%	44%
Immigrant background, %	4.8%	4.4%	6.8%	7.8%	4.1%	5.9%
Number of observations, N	23,824	69,278	64,905	76,335	44,277	278,619
Share of observations, %	9%	25%	23%	27%	16%	100%

^1^ The child entered day care right after the parental leave period ended at approximately 10 months old.

**Fig 3:**
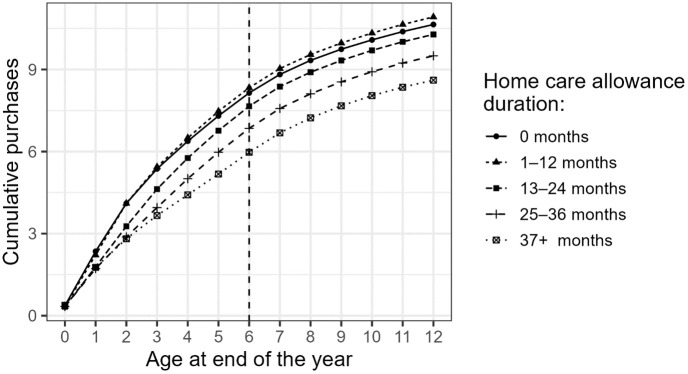
The average cumulative number of antibiotic purchases 0–12 years after birth by the duration of home care allowance.

In Fig 3, we show the average cumulative number of antibiotic purchases during the child’s first 12 years by the duration of HCA. The descriptive findings show that the higher the number of months in home care, implying later entry to day care, the lower the number of average cumulative antibiotic purchases. During the entire examined period, the average number of cumulative antibiotic purchases remains lowest in the group of children with the longest home care periods. In children who were in home care for 1–12 months (until approximately 1–2 years of age) or entered day care right after the parental leave period ended (approximately 10 months of age), the cumulative antibiotic use remains at the highest level throughout the follow-up period.

**Fig 4:**
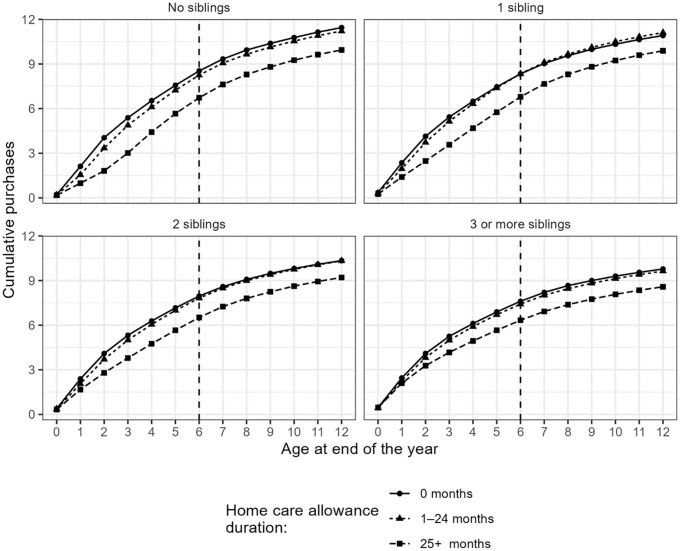
The average cumulative number of antibiotic purchases 0–12 years after birth by the duration of home care allowance and number of siblings.

In Fig 4, we show the average cumulative number of antibiotic purchases 0–12 years after birth by the duration of HCA (until the child turns 6 years old) and number of siblings. The number of cumulative antibiotic purchases remains lowest in the group of children with the longest HCA duration, regardless of the number of siblings. However, in all HCA duration categories, the average cumulative number of antibiotic purchases was lowest in children with three or more siblings.

### Results from the Poisson regression model

Next, we show that accounting for the mother’s characteristics or the presence of older siblings using statistical methods does not greatly affect the findings of our descriptive analyses. Table 2 presents findings from the unadjusted model (Model 1) as well as from the model accounting for mothers’ education and age at childbirth and a dummy if the child is the second or subsequent child (Model 2). Furthermore, Table 2 (unadjusted model, Panel A, constant line) shows that between the ages 0–12, children in the sample have 10.7 antibiotic purchases, on average.

**Table 2:** Cumulative antibiotic purchases between the ages of 0 and 12 by the duration of home care allowance.

	Unadjusted	Adjusted
	(Model 1)	(Model 2)
	IRR	IRR
**Home care allowance (HCA) duration**		
13–24 months of HCA	1	1
0 months – no HCA^1^	1.033***	1.026***
1–12 months of HCA	1.062***	1.054***
25–36 months of HCA	0.922***	0.932***
37+ months of HCA	0.835***	0.835***
**Controls**		
Full controls		X
Exp(Constant)	10.689***	9.532***

^1^ The child entered day care right after the parental leave period ended at approximately 10 months old.

Child’s birth year included in all specifications. Adjusted model includes controls for mother’s immigrant background, education, and age at childbirth and a dummy if the child has older siblings. Number of observations N = 278,619. *p < 0.1; **p < 0.05; ***p < 0.01. IRR = Incidence Rate Ratio

The results from the Poisson model, expressed in incidence rate ratios (IRRs), further affirm that the cumulative use of antibiotics differs between the HCA duration groups and that the cumulative use is lower in children with longer HCA duration.

Compared to the reference group (HCA for 13–24 months), higher expected number of antibiotic purchases is associated with groups with shorter HCA duration (IRR > 1), and lower cumulative use with groups with longer HCA duration (IRR < 1), regardless of maternal characteristics or the presence of older siblings.

Children with an HCA duration of 37 months or longer had, on average, 1.6 fewer antibiotic purchases than the children in the reference group in the adjusted model.

Lastly, we examine the association between the number of siblings and HCA duration, as older children are often in home care alongside their younger sibling. Table 3 divides the sample by the number of biological siblings at age 12. The results remain consistent with those in Table 2 and Fig 3. Table 3 also shows that children who enter day care earlier, i.e., whose HCA duration was shorter, have more antibiotic purchases between the ages of 0 and 12 when compared to children who entered day care later, i.e., have longer home care periods. Again, accounting for maternal characteristics or the existence of older siblings does not change the direction of the association.

**Table 3:** Cumulative antibiotic purchases between the ages of 0 and 12 by number of siblings and duration of home care. Fully adjusted models.

	Single child	1 sibling	2 siblings	3 or more siblings
	(Model 1)	(Model 2)	(Model 3)	(Model 4)
	IRR	IRR	IRR	IRR
**Home care allowance (HCA) duration**				
13–24 months of HCA	1	1	1	1
0 months – no HCA	1.044***	1.006*	1.013***	1.043***
1–12 months of HCA	1.065***	1.051***	1.051***	1.057***
25–36 months of HCA	0.933***	0.939***	0.928***	0.948***
37 + months of HCA	0.974	0.870***	0.850***	0.867***
Exp(Constant)	9.237***	9.928***	9.319***	8.842***
N	19,640	98,627	86,928	73,424

Child’s birth year included in all specifications. Number of siblings corresponds to mother’s number of biological children at the end of the follow-up period (t = 12 when the reference child’s age is 12). All model includes controls for mother’s immigrant background, education, and age at childbirth and a dummy if the child has older siblings*p < 0.1; **p < 0.05; ***p < 0.01. IRR = Incidence Rate Ratio

Supplementary results by gender, region and time period are provided in the S1-S3 Tables. Briefly, boys have more antibiotics purchases than girls (9.96 purchases vs. 9.07 purchases), and children born in cities had larger cumulative purchases when compared children born in rural areas. However, the results on HCA duration remain consistent with Table 2 and Table 3, with a few minor exceptions. Children who reside in rural areas and spend long periods in home care made fewer purchases compared to children living in urban areas with the same duration in home care. There were no large differences in cumulative antibiotic use at age 12 between different birth cohorts (cohorts 2000–2006 in this study) but the connection between long HCA duration and cumulative purchases grew stronger for those in later birth cohorts.

## Discussion

The results of our study show that almost all children were exposed to prescribed antibiotics within the first 12 years of their life. One fifth of the children had more than 15 antibiotic purchases by the age of 12. The three most commonly purchased antibiotics for 0–12-year-old children between 2000 and 2017 were amoxicillin, azithromycin, and cefalexin. These medicines are often prescribed for children to treat bacterial ear and chest infections and tonsillitis, for example. It is noteworthy that azithromycin, the second most commonly used antibiotic, belongs to the macrolide class, which is generally not among the most recommended first-line treatment options according to current guidelines. The overuse of broad-spectrum macrolide antibiotics in children has also been observed in previous studies [5,39].

On average, the children had seven antibiotic purchases by the end of the year they turned 6 years and ten antibiotic purchases by age 12. The cumulative number of purchases was lowest in the groups of children whose home care period (before school age) was the longest. The differences between the groups in the average cumulative number of antibiotic purchases largely persisted until the age of 12. These findings suggesting that lower cumulative antibiotic exposure is associated with longer home care duration remained robust even after controlling for maternal characteristics and the number of siblings.

Earlier studies have shown that the risk of respiratory tract infections is higher among children in day care because they are more recurrently exposed to acute respiratory infections than children in home care [21,40]. Moreover, it is known that there is a rapid increase in sick days during the first few months after entering day care [22] and that earlier day care entry is associated with more antibiotic use in childhood and adolescence [23]. However, to our knowledge, this is one of the first register-based study investigating the association between antibiotic purchases and the duration of home care over an extended period of time, i.e., until the children are 12 years old.

Our findings show that the shorter the HCA period, i.e., the earlier the child enters day care, the more antibiotic purchases the child has at the end of the follow-up when she or he is 12 years old compared to the reference group. For example, those with HCA duration of 37 months or longer, had on average, 1.6 fewer antibiotic purchases than those in the reference group. Moreover, most of the differences in antibiotic use between different HCA groups can be attributed to the first 6 years after birth; antibiotic use remains relatively stable between ages 6 and 12 among children with different HCA duration. Though the clinical relevance of 1.6 purchases during 12 years of follow-up can be debated, it may still reflect meaningful variation in exposure to infections and health care use over time. Even relatively small differences in antibiotic use at the population level can be important from a public health perspective, particularly in the context of antimicrobial resistance and stewardship efforts [3].

We found the overall cumulative number of antibiotic purchases is lower among children with a higher number of siblings. The cumulative antibiotic exposure was lowest among children who spent 25 months or longer in home care and who had three or more siblings and highest among those with no siblings and no home care period. In the Poisson regression analyses, we also found that the presence of older siblings is associated with a lower cumulative use of antibiotics. Toivonen et al. (2016) [19] have shown that having older siblings could increase the risk of recurrent infections in small children. However, the severity of infections may vary and not all infections require antibiotic treatment.

The strength of our study is that we used high-quality register data based on a nationally representative sample of children and families covering 80% of all live births in Finland. Our follow-up time was exceptionally long compared to most of the earlier studies on the association of childcare and antibiotics, as we followed the children’s antibiotic purchases from birth to when they were 12 years old. We were also able to apply individual-level information on HCA use to deduce a reverse measure for day care attendance [34,35], as national individual-level information on day care attendance before the 2020s are unavailable in many countries. Finally, many previous studies on day care attendance and antibiotic use have relied on small samples, limited geographical coverage, or questionnaire-based data, rather that large-scale register data.

Despite the considerable strengths, our study also has limitations. Using HCA as a proxy for day care attendance may oversimplify children’s care arrangements and potentially introduce misclassification bias. For instance, some children who do not attend formal day care may receive informal care, which could increase their exposure to respiratory tract infections. However, such informal care arrangements are relatively uncommon in Finland [33], which strengthens the validity of our measure. In addition, families with multiple children are slightly over-represented in our sample. There may also be confounding factors, such as parental behaviour, affecting both day care entry and antibiotic utilisation, that we were not able to account for. During the study period 2000–2017, the outpatient prescribing practices for antibiotics changed, leading to a decrease in the overall prescribing of antibiotics in Finland [6,7]. Though we use child’s birth year in our models, some differences related to the observation time might elude this correction. In addition, the COVID-19 pandemic, known to impact antibiotic prescribing also in Finland [8], is beyond the time frame of our study. This may hinder the generalisability of our findings. Furthermore, our data on antibiotics did not cover all antibiotics used in outpatient care because not all of them were included in the list of reimbursable medicines at the time of the study [6]. This means that our estimate for the total number of antibiotic purchases is likely an underestimation, as it lacks information on non-reimbursed purchases. However, we assume that the range of prescribed and purchased antibiotics was similar in all studied groups, and that the differences in reimbursed antibiotic purchases between the studied groups reflect the differences in all antibiotic purchases.

Future research should examine the total number of antibiotic purchases, including those not reimbursed, with particular emphasis on the types and spectrum of antibiotics prescribed. Special attention should be given to broad-spectrum antibiotics, which are more likely to contribute to antimicrobial resistance and therefore warrant closer monitoring in both research and policy contexts.

In addition to further research, strategies to minimise the spread of infectious diseases in day care settings should be prioritised. Measures such as good hand hygiene, regular cleaning and disinfecting, and proper ventilation, and smaller group sizes have been shown to reduce transmission, especially during and after the COVID-19 pandemic [41,42]. Lowering the incidence of infections and reducing antibiotic use in early childhood care environments could yield both clinical and environmental benefits [43], and support broader public health and antimicrobial stewardship goals.

## Conclusions

We show that the average cumulative number of antibiotic purchases from birth to age 12 is lowest among children with the longest duration of home care. The number of siblings does not appear to influence the outcome, and the results remain robust even after controlling for socioeconomic status. Although the differences between groups are moderate, they may still have meaningful implications at the population level, particularly in the context of antimicrobial resistance and public health. These findings, based on large-scale register data, contribute to a broader understanding of how early childhood care arrangements may be associated with long-term patterns of antibiotic use.

## Supporting information

S1 Table Cumulative antibiotic purchases between the ages of 0 and 12 by gender and duration of home care. Fully adjusted model.
(DOCX)

S2 Table Cumulative antibiotic purchases between the ages of 0 and 12 by home address’ urban-rural classification and duration of home care. Fully adjusted model.
(DOCX)

S3 Table Cumulative antibiotic purchases between the ages of 0 and 12 by birth year and duration of home care. Fully adjusted model.
(DOCX)
