# Supplementary material for: The impact of day care attendance on antibiotic use among children aged 0–12 years: A population-based register study
Source: PLoS One. 2025 Nov 17;20(11):e0335354. doi: 10.1371/journal.pone.0335354 (PMC12622847; doi:10.1371/journal.pone.0335354)
Supplement: S1 Table — Fully adjusted model. (DOCX) [file pone.0335354.s001.docx]

**S1 TABLE: GENDER.** Cumulative antibiotic purchases between the ages of 0 and 12 by gender and duration of home care. Fully adjusted model.

|  | Boys | Girls |
| --- | --- | --- |
|  | (1) | (2) |
|  | IRR | IRR |
| **Home care allowance (HCA) duration** |  |  |
| 13–24 months of HCA | 1 | 1 |
| 0 months - no HCA | 1.021*** | 1.031*** |
| 1–12 months of HCA | 1.049*** | 1.060*** |
| 25–36 months of HCA | 0.933*** | 0.931*** |
| 37+ months of HCA | 0.841*** | 0.830*** |
| Exp(Constant) | 9.955*** | 9.065*** |
| N | 142,919 | 135,700 |

Notes: Control for child’s birth year included in all specifications. Adjusted model includes controls for mother’s immigrant background, education, and age at childbirth and a dummy if the child has older siblings. *p<0.1; **p<0.05; ***p<0.01. IRR=Incidence Rate Ratio.
