# Supplementary material for: The impact of day care attendance on antibiotic use among children aged 0–12 years: A population-based register study
Source: PLoS One. 2025 Nov 17;20(11):e0335354. doi: 10.1371/journal.pone.0335354 (PMC12622847; doi:10.1371/journal.pone.0335354)
Supplement: S2 Table — Fully adjusted model. (DOCX) [file pone.0335354.s002.docx]

**S2 TABLE: REGION: Urban-rural.** Cumulative antibiotic purchases between the ages of 0 and 12 by home address’ urban-rural classification and duration of home care. Fully adjusted model.

|  | K1-K3  Urban | M4  Rural | M5  Rural | M6  Rural | M7  Rural |
| --- | --- | --- | --- | --- | --- |
|  | (1) | (2) | (3) | (4) | (5) |
|  | IRR | IRR | IRR | IRR | IRR |
| **Home care allowance (HCA) duration** |  |  |  |  |  |
| 13–24 months of HCA | 1 | 1 | 1 | 1 | 1 |
| 0 months - no HCA | 1.060*** | 0.945*** | 1.019** | 0.952*** | 1.008 |
| 1–12 months of HCA | 1.059*** | 1.059*** | 1.035*** | 1.032*** | 1.029*** |
| 25–36 months of HCA | 0.941*** | 0.941*** | 0.910*** | 0.927*** | 0.901*** |
| 37+ months of HCA | 0.833*** | 0.860*** | 0.827*** | 0.927*** | 0.827*** |
| Exp(Constant) | 9.628*** | 9.086*** | 9.790*** | 8.897*** | 8.789*** |
| N | 194,458 | 16,209 | 20,925 | 31,982 | 13,948 |

Notes: Control for child’s birth year included in all specifications. Adjusted model includes controls for mother’s immigrant background, education, and age at childbirth and a dummy if the child has older siblings. Table uses urban-rural classification from Statistics Finland with the following groups in columns (1) to (7) used: K1–K3 = urban area, M4 = Local centres in rural areas, M5 = Rural areas close to urban areas, M6 = Rural heartland areas, and M7 = Sparsely populated rural areas. See <https://stat.fi/meta/kas/kaupunki_maaseu_en.html> for the full definition. *p<0.1; **p<0.05; ***p<0.01. IRR=Incidence Rate Ratio.
